# Effects of Clay Nanosheets on the Photostability of Cationic Porphyrin

**DOI:** 10.3390/molecules29163738

**Published:** 2024-08-07

**Authors:** Yoshinori Tahara, Yugo Hirade, Kyosuke Arakawa, Tetsuya Shimada, Tamao Ishida, Hiroshi Tachibana, Shinsuke Takagi

**Affiliations:** 1 Department of Applied Chemistry, Faculty of Urban Environmental Sciences, Tokyo Metropolitan University, Hachiohji, Tokyo 192-0397, Japan; 2 Advanced Collaborative Research Organization for Smart Society (ACROSS), Waseda University, Shinjuku-ku, Tokyo 169-8555, Japan; 3 Department of Applied and Pure Chemistry, Faculty of Science and Technology, Tokyo University of Science, Noda-City 278-8510, Chiba, Japan; 4 Research Center for Hydrogen Energy-Based Society (ReHES), Tokyo Metropolitan University, Hachiohji, Tokyo 192-0397, Japan

**Keywords:** absorption spectroscopy, clay, photostability

## Abstract

The photodecomposition behavior of cationic porphyrin ZnTMAP^4+^ (zinc tetrakis-(*N*,*N*,*N*-trimethylanilinium-4-yl) porphyrin) in water and complexed with clay nanosheets was investigated by light irradiation to the Soret band of ZnTMAP^4+^. The decomposition of ZnTMAP^4+^ was observed by UV–visible absorption spectroscopy. While the decomposition quantum yield (*ϕ*_dec_) was 3.4 × 10^−4^ in water, that was 9.4 × 10^−7^ on the exfoliated clay nanosheets. It was revealed that the photostability of ZnTMAP^4+^ was stabilized by the complex formation with clay. When ZnTMAP^4+^ was intercalated between the stacked clay nanosheets, *ϕ*_dec_ was further decreased to 4.9 × 10^−7^. The photostability increased by 361 times and 693 times for the exfoliated and stacked state, respectively. These results indicate that the flat clay surface has the potential to control intra- and intermolecular photochemical reactions.

## 1. Introduction

Clay minerals are inorganic layered compounds with extremely flat surfaces. Saponite, one of the smectite clay minerals, has a negatively charged surface and can be dispersed and exfoliated in water [1,2,3]. It can incorporate various substances via electrostatic and hydrophobic interactions, and its transparency in the UV–visible region makes it suitable for constructing photochemical reaction systems and evaluating photochemical properties of chemical species that have been absorbed on clay nanosheets [4,5,6,7,8,9,10].

While the durability of organic dyes is an issue from the viewpoint of materials sciences, the decomposition and removal of organic dyes are subjects from the viewpoint of environmental sciences. The complex formation of dyes with host materials could affect the photophysical property and stability of dyes. For example, V. Ramamurthy et al. reported that zeolites play an important role in organic photochemistry [11], and George S. Hammond et al. examined medium effects on photochemical reactions [12]. For example, it was reported that zeolites can control the outcome of photoreactions, allowing control and prediction of the type and properties (regiochemical and stereochemical features) of the products [11].

It has been reported that organic dyes composited with clay nanosheets are stabilized [11,12,13,14,15,16,17,18] or destabilized [19,20,21,22]. Our group has reported the effects of clay nanosheet surfaces on “intra”-molecular processes and reactions, such as luminescence enhancement (Surface-Fixation Induced Emission: s-FIE) and control of trans cis photoisomerization, in clay–dye complexes [23,24,25,26,27]. In addition, we recently reported the effects of the clay nanosheet surface on “inter”-molecular chemical reactions, and showed that not only the electronic effect but also the steric effect of clay surface plays an important role in chemical reactions [28]. Thus, the flat surface of clay nanosheets is expected to work as a reaction field to control “intra”- and “inter”-molecular chemical reactions and thus alters the photostability of dyes. In the case of specific dyes such as tetra cationic porphyrins, it is known that dyes do not form aggregates on the clay surface, because of the size-matching effect [29,30]. The photochemical chlorination and oxygenation reaction of cyclohexene sensitized by a Ga(III) porphyrin–clay mineral system was reported [31]. In the reaction system, the decomposition of Ga(III) porphyrin was suppressed by the anionic clay surface sterically and electrically. Thus, tetra cationic porphyrin is a suitable dye to investigate the photochemical behavior on clay surfaces.

**Figure 1:**
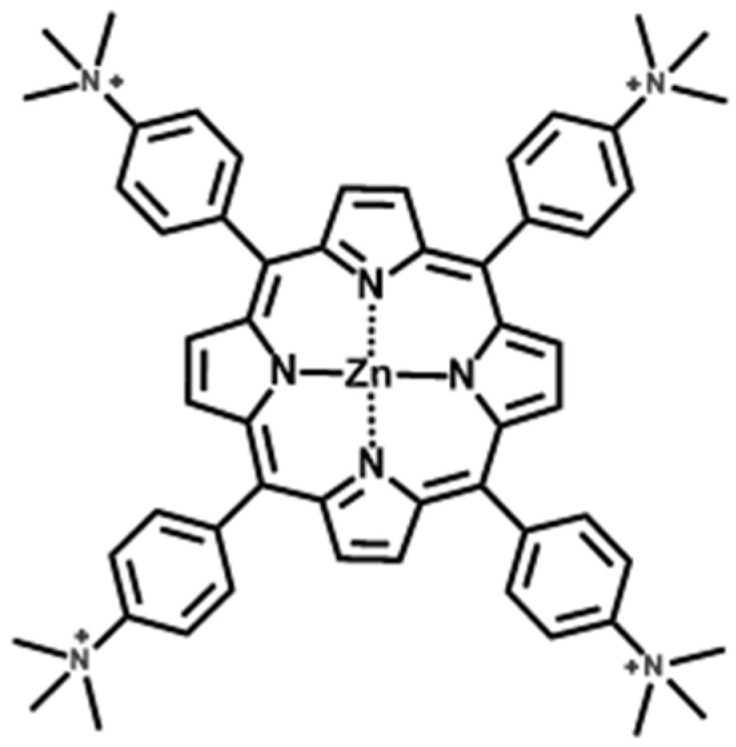
The structure of ZnTMAP^4+^.

Therefore, the purpose of this research is to improve the photostability of a typical functional dye, zinc tetrakis-(*N*,*N*,*N*-trimethylanilinium-4-yl) porphyrin (ZnTMAP^4+^, Figure 1), by focusing on the flatness of clay nanosheets at the atomic level. The results of this research are expected to be applied not only to color materials but also to basic research on photochemical reactions such as energy and electron transfer on clay nanosheets for artificial photosynthesis.

## 2. Results and Discussion

### 2.1. Decomposition Behavior of ZnTMAP^4+^ in Water and on Exfoliated Clay under Light Irradiation

**Figure 2:**
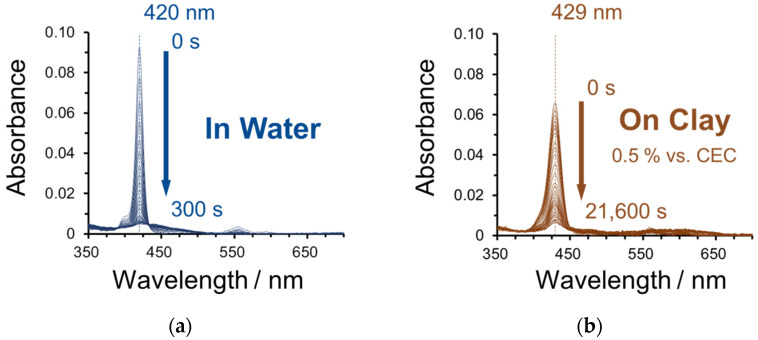
Absorption spectra of ZnTMAP^4+^ at each light irradiation time (**a**) in water and (**b**) on clay (0.5% vs. CEC). Light irradiation: 160 mW single-color LED lamp (in water 415 nm, on clay 430 nm). Concentration of dye is 2.66 × 10^−7^ M and clay is 5.32 × 10^−9^ eq L^−1^. The loading level of ZnTMAP^4+^ is 0.5% vs. the CEC of the clay.

The absorption spectra of ZnTMAP^4+^ in water and in a clay dispersion during the light irradiation are shown in Figure 2. The loading level of ZnTMAP^4+^ was set at 0.5% vs. the CEC (Cation Exchange Capacity) of the clay. The maximum absorption wavelengths before light irradiation were 420 nm and 429 nm for “in water” and “on clay” samples, respectively. It is known that the absorption *λ*_max_ of the Soret band of cationic porphyrins complexed with saponite shifts to the longer wavelength side with the co-planarization of the porphyrin ring and peripheral aromatic rings at the *meso* position [23]. Under the present conditions, it is known that ZnTMAP^4+^ can achieve high-density adsorption without aggregation on clay nanosheets, and all of the ZnTMAP^4+^ in this experimental system is adsorbed on clay nanosheets without aggregation [30]. The absorbance at the Soret band almost disappeared after 300 s of light irradiation for the “in water” sample, whereas it did not disappear completely after 21,600 s of light irradiation for the “on clay” sample, indicating that ZnTMAP^4+^ is more stable to the light on the clay nanosheet than in water.

**Figure 3:**
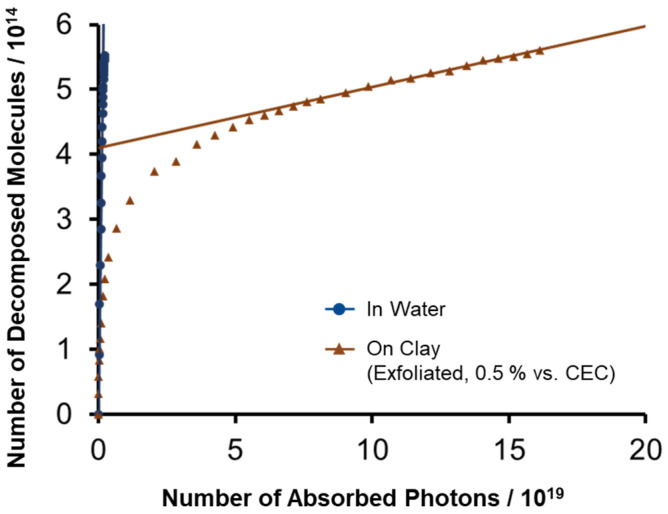
Photodecomposition behavior of ZnTMAP^4+^ in water and in exfoliated clay dispersion. Concentration of dye is 2.66 × 10^−7^ M and clay is 5.32 × 10^−9^ eq L^−1^. The loading level of ZnTMAP^4+^ is 0.5% vs. the CEC of the clay.

The photodecomposition behavior of ZnTMAP^4+^ in water and on clay nanosheets is shown in Figure 3. The horizontal and vertical axes are the number of absorbed photons and the number of decomposed molecules, respectively. In this graph, the slope corresponds to the decomposition quantum yield. The photodegradation of ZnTMAP^4+^ in water seems to be a pseudo-first-order reaction, since it shows a linear change for most of the irradiation period, although the decomposition process includes multi steps after the breakage of the porphyrin macrocycle [32]. The enlarged graph is shown in Figure S1. From the slope, the decomposition quantum yield for the “in water” sample is *ϕ*_dec_ = 3.4 × 10^−4^. The photodecomposition of ZnTMAP^4+^ on clay nanosheets did not show a linear change. This means that the decomposition of porphyrins is not a simple first-order reaction, or that there are multiple porphyrin adsorbed species. At the later stages of the photoreaction (>7 × 10^19^ photons), the slope became linear. It is known that the adsorbed species is not uniformly adsorbed and thus is concentrated on the clay surface [33]. ZnTMAP^4+^ has also been reported as a self-quenching molecule on clay nanosheets [34]. Based on the above, it is likely that the photodecomposition of ZnTMAP^4+^ on clay nanosheets involves an intermolecular reaction between ZnTMAP^4+^ molecules [35]. On the other hand, two different orientations of the porphyrin, that is, parallel and vertical to the clay surface, could lead to the two components’ decomposition behavior. It is known that the absorption maximum of porphyrin strongly depends on the adsorption orientation [23]. Judging from the observed absorption maximum, all porphyrin is adsorbed on the clay surface in a parallel way. Thus, it is supposed that the intermolecular decomposition proceeds in the early stage of the reaction (<7 × 10^19^ photons), while the unimolecular decomposition of porphyrins proceeds in the late stage of the reaction (>7 × 10^19^ photons), where the existence density of ZnTMAP^4+^ becomes low. Therefore, the *ϕ*_dec_ of a single ZnTMAP^4+^ molecule on a clay nanosheet could be obtained from the slope of a straight line drawn in the late reaction region, and a value of *ϕ*_dec_ = 9.4 × 10^−7^ was obtained. This value indicates that ZnTMAP^4+^ on clay nanosheets is 361 times more light-stabilized than in water in the monomolecular state. Intermolecular reactions are considered to be caused by collision between excited dye molecules and ground-state dye molecules, such as an electron transfer.

### 2.2. Effect of Adsorption Density of ZnTMAP^4+^ on Decomposition Behavior under Light Irradiation

**Figure 4:**
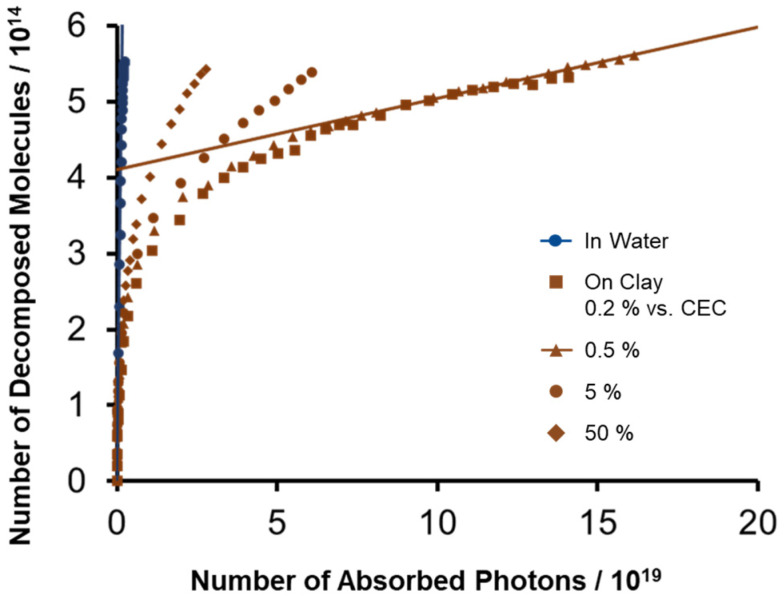
Effects of adsorption density on the photodecomposition behavior of ZnTMAP^4+^. Concentration of dye is 2.66 × 10^−7^ M and clay is 2.13 × 10^−9^ eq L^−1^, 5.32 × 10^−9^ eq L^−1^, 5.32 × 10^−8^ eq L^−1^, and 5.32 × 10^−7^ eq L^−1^. The loading level of ZnTMAP^4+^ is 0.2, 0.5, 5, and 50% vs. the CEC of the clay.

The adsorption density of ZnTMAP^4+^ on clay nanosheets was varied (0.2, 0.5, 5, and 50% vs. the CEC of the clay). The photodecomposition behavior of these samples is shown in Figure 4. Photodecomposition of ZnTMAP^4+^ on clay nanosheets was more likely to occur at higher molecular densities and less likely to occur at lower densities, indicating that intermolecular reactions between dyes are involved in the decomposition process. In the later stage of the photoreaction, the decomposition behavior of the samples with adsorption densities of 0.2% and 0.5% vs. the CEC was almost identical. In other words, in this region, decomposition due to intermolecular reactions between dyes is negligible, indicating that degradation proceeds purely as a single molecule. The good match between the slopes of the linear lines at the later stage of the reaction at 0.2% and 0.5% adsorption confirms that the value of *ϕ*_dec_ = 9.4 × 10^−7^ obtained at 0.5% adsorption is the value for the unimolecular photodecomposition reaction of ZnTMAP^4+^ on the clay.

### 2.3. Decomposition Behavior of ZnTMAP^4+^ between the Stacked Clay Layers 

**Figure 5:**
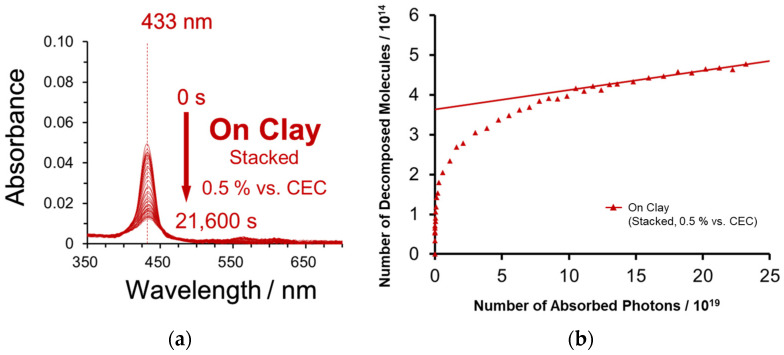
(**a**) Absorption spectra of ZnTMAP^4+^ complexed with stacked clay nanosheets at each light irradiation time and (**b**) comparison of photodecomposition behavior of ZnTMAP^4+^ complexed with stacked clay nanosheets. Concentration of dye is 2.66 × 10^−7^ M and clay is 5.32 × 10^−9^ eq L^−1^. The loading level of ZnTMAP^4+^ is 0.5% vs. the CEC of the clay.

In the previous section, the photodecomposition behavior of dyes adsorbed on the exfoliated nanosheets was investigated. On the other hand, nanosheets can stack and further stabilization against light [15] is expected in ZnTMAP^4+^ intercalated between the stacked nanosheets. Thus, the photodegradation behavior of ZnTMAP^4+^ between the stacked clay layers was examined. The intercalation sample was prepared by the freeze–thaw method [30]. The absorption spectral change and photodecomposition behavior of ZnTMAP^4+^ intercalated between stacked clay nanosheet layers at 0.5% vs. the CEC with light irradiation are shown in Figure 5. The *λ*_max_ of ZnTMAP^4+^ composited with the stacked clay nanosheet is shifted to a longer wavelength (433 nm) compared to that of the exfoliated clay, indicating that the molecule exists in a more planarized state [23]. As can be seen in Figure 5b, the stability of ZnTMAP^4+^ was increased by the intercalation compared to that of the exfoliated clay. The value of *ϕ*_dec_ for ZnTMAP^4+^ intercalated between the clay nanosheet layers was determined to be 4.9 × 10^−7^ based on the slope of the late photoreaction phase (Figure 5b straight line). This value for the intercalated sample was 693 times more stabilized than in water and 1.9 times more stabilized than in the exfoliated one (Table 1). The stabilizing effect is thought to be due to the fact that the molecules are covered, which hinders the approach of reactive substrates that contribute to photodegradation, and also because they are fixed in a more planar structure.

**Figure 6:**
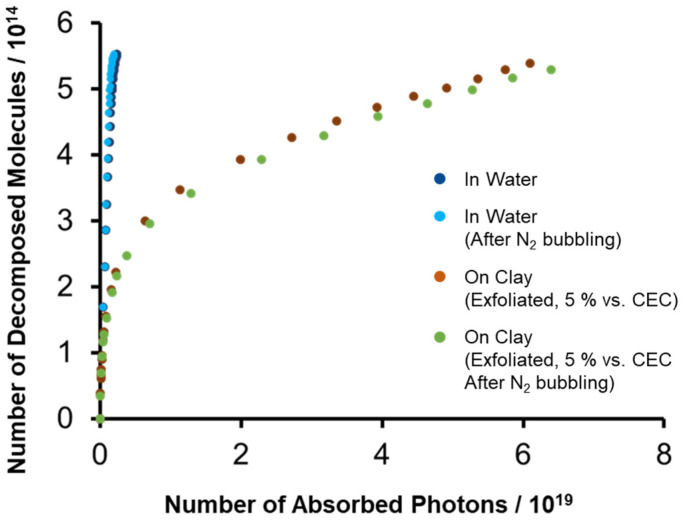
Effects of oxygen on the photodecomposition behavior of ZnTMAP^4+^.

**Figure 7:**
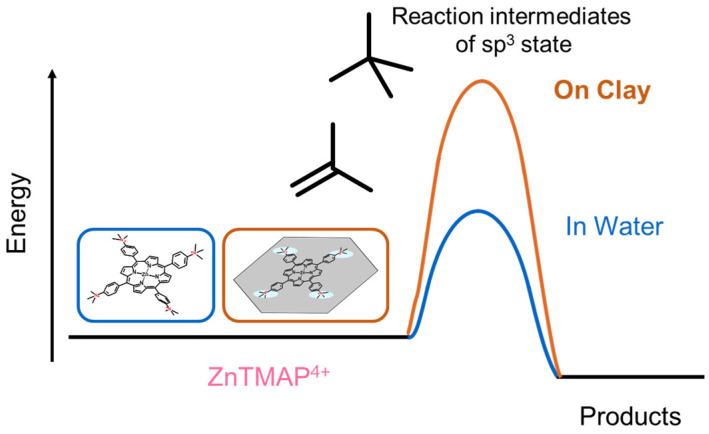
Image of the possible effect of the clay surface on the potential energy surface for the decomposition of porphyrin.

The possible decomposition mechanisms of porphyrin are (i) unimolecular decomposition, (ii) decomposition by an attack of a substrate such as water molecules, (iii) degradation by an attack of oxygen molecules. Mechanism (iii) seems not to be a major factor, since the decomposition behavior was not affected when the reaction was carried out under nitrogen bubbling for both case with and without the clay (Figure 6). The concentration of substrates is expected to be around half, since half of the ZnTMAP^4+^ is covered by the clay nanosheet. Because the decrease in *ϕ*_dec_ due to adsorption on the clay is significant, as shown in Table 1, decomposition mechanism (ii) due to the simple covering effect of clay is unlikely to be the main mechanism. Therefore, it is presumed that the effect of the clay surface on the photodegradation of ZnTMAP^4+^ is mainly due to the suppression of mechanism (i) and/or (ii), not due to the simple covering effect of clay. In the photodecomposition process of ZnTMAP^4+^, sp^2^ carbon at the *meso* position could be attacked by substrates and sp^3^ carbon could form as the reaction intermediate [31,35]. It was reported that the decomposition of Ga(III) porphyrin through its cation radial was suppressed by the anionic clay surface sterically and electrically to prevent the formation of sp^3^ carbon. Thus, on clay nanosheets, it is speculated that the molecules are fixed in a planar structure, which increases the activation energy to generate the sp^3^ carbon, thus suppressing the progress of the photodecomposition reaction (Figure 7).

## 3. Materials and Methods

### 3.1. Chemicals

Zinc tetrakis-(*N*,*N*,*N*-trimethylanilinium-4-yl) porphyrin (ZnTMAP^4+^) chloride (4Cl^−^) was purchased from Frontier Scientific Inc., (Newark, DE, USA). Sumecton SA (SSA) as a synthetic saponite was received from Kunimine Industries Co., Ltd (Tokyo, Japan). The stoichiometric formula of SSA is [(Si_7.20_Al_0.80_)(Mg_5.97_Al_0.03_)O_20_(OH)_4_]^−0.77^(Na_0.49_Mg_0.14_)^+0.77^. The cation exchange capacity (CEC) of the saponite is ca. 1.0 × 10^−3^ equiv. g^−1^. The CEC value was calculated using the formula weight and the number of negative charges in the formula. The average distance between the anionic points on the saponite surface is estimated to be 1.2 nm, on the basis of the assumption of a hexagonal array. While natural clay often includes transition metals such as Fe^3+^ in the structure and perturb the excited state of adsorbed dyes, synthetic clay is inactive from the viewpoint of redox reactions.

### 3.2. Preparation of Clay–Dye Complexes

Aqueous stock solutions of SSA and ZnTMAP^4+^ were mixed under stirring. The typical concentration of ZnTMAP^4+^ was 2.66 × 10^−7^ M. The loading level of ZnTMAP^4+^ was controlled by changing the concentration of the clay dispersion. Under the conditions, it was confirmed that all ZnTMAP^4+^ was adsorbed on the clay surface [29,30]. The loading level of porphyrin was defined as “number of positive charges due to ZnTMAP^4+^/number of negative charges due to the clay in the system”.

The samples where ZnTMAP^4+^ was intercalated by clay nanosheets were prepared by repeating four times a freeze (liquid N_2_)–thaw (hot water) cycle with exfoliated complexes [29,30]. It should be noted that the clay dispersion is substantially transparent because the particle size of the clay nanosheet is around 30 nm with a 0.96 nm thickness. The particle size was observed by AFM and DLS. The thickness is a theoretical value, and a similar value was observed by AFM. A typical AFM image can be seen in ref. [29].

For the experiment examining oxygen effect, the PMMA (polymethylmethacrylate) cell containing the sample solution was sealed using silicon rubber and sealing tape, and nitrogen bubbling was performed for 30 min just before light irradiation.

### 3.3. Light Irradiation

An LED lamp (THOLABO Japan (Tokyo, Japan)) at 415 nm (in water) or 430 nm (in clay dispersion) was used as the excitation light source. An amount of 4.0 mL of sample solution was irradiated in a PMMA cell with a 1 cm optical path length with stirring. The intensity of the LED lamp was adjusted to 160 mW. Because cationic porphyrin tends to be adsorbed onto glass, a PMMA cell was used to avoid the adsorption of porphyrin.

### 3.4. Estimation of the Number of Absorbed Photons

The number of irradiated photons was determined for each wavelength region divided from the emission spectrum of the LED lamp measured by the USB spectrometer and the irradiation light intensity measured by the power meter (ADC (Saitama, Japan), 8230E). This was multiplied by the absorption spectrum for each irradiation time in the entire wavelength range to estimate the number of absorbed photons.

### 3.5. Photodecomposition Quantum Yield

The photostability of the dye was evaluated by the quantum yield of the decomposition defined by Equation (1). The number of decomposed porphyrin molecules was estimated by the absorption change during the irradiation and the absorption coefficient of ZnTMAP^4+^.

(1) $$\phi_{dec}=\frac{Number\;of\;Decomposed\;Molecules}{Number\;of\;Absorbed\;Photons}$$

## 4. Conclusions

The photodecomposition behavior of ZnTMAP^4+^ was examined in water, on clay surfaces, and between clay nanosheets. The number of decomposed molecules was plotted against the number of absorbed photons. For the “in water” sample, a constant slope was observed during the longest period of irradiation. For the “on clay” and “between clay” samples, a constant slope was observed at a later period of the irradiation. As a whole, the photodecomposition of ZnTMAP^4+^ was suppressed when ZnTMAP^4+^ was adsorbed on or intercalated by clay nanosheets, compared to the “in water” sample. From the value of the constant slope for each sample, the decomposition quantum yield (*ϕ*_dec_) was calculated. While *ϕ*_dec_ was 3.4 × 10^−4^ in water, that for the exfoliated and intercalated samples was 9.4 × 10^−7^ and 4.9 × 10^−7^, respectively. It is presumed that the effect of the clay surface on the photodegradation of ZnTMAP^4+^ is mainly due to the suppression of unimolecular decomposition and/or intermolecular reactions. On clay nanosheets, it is speculated that the molecules are fixed in a planar structure, which increases the activation energy to generate the sp^3^ carbon, thus suppressing the progress of the photodecomposition reaction. This research indicates that host materials can play an important role for photo-chemical and physical processes, as Prof. Ramamurthy et al. claimed [7,11,36]. Because synthetic clay minerals have many characteristics, such as a flat surface and negative charge array, they could extend the possibility of photochemistry to resolve environmental and energy problems.

## Figures and Tables

**Table 1:** Decomposition quantum yield of ZnTMAP^4+^ in water without and with clay.

ZnTMPyP^4+^	*ϕ*_dec_/10^−7^
Water	3400
Clay (Exfoliated)	9.4
Clay (Stacked)	4.9

## Data Availability

The original contributions presented in the study are included in the article (and Supplementary Materials), further inquiries can be directed to the corresponding authors.

## Supplementary Materials

The following supporting information can be downloaded at: https://www.mdpi.com/article/10.3390/molecules29163738/s1, Figure S1: Photodecomposition behavior of ZnTMAP^4+^ in water and in exfoliated clay dispersion.

## References

[1] Li C., Wu Q., Petit S., Gates W.P., Yang H., Yu W., Zhou C. (2019). Insights into the Rheological Behavior of Aqueous Dispersions of Synthetic Saponite: Effects of Saponite Composition and Sodium Polyacrylate. Langmuir.

[2] Dazas B., Lanson B., Delville A., Robert J.L., Komarneni S., Michot L.J., Ferrage E. (2015). Influence of Tetrahedral Layer Charge on the Organization of Interlayer Water and Ions in Synthetic Na-Saturated Smectites. J. Phys. Chem. C.

[3] Vogels R.J.M.J., Kloprogge J.T., Geus J.W. (2005). Synthesis and characterization of saponite clays. Am. Miner..

[4] Ogawa M., Kuroda K. (1995). Photofunctions of Intercalation Compounds. Chem. Rev..

[5] Suzuki Y., Tenma Y., Nishioka Y., Kawamata J. (2012). Efficient Nonlinear Optical Properties of Dyes Confined in Interlayer Nanospaces of Clay Minerals. Chem.-Asian J..

[6] Yamagishi A., Tamura K., Yamamoto S., Sato F., Yoshida J. (2024). Up-conversion of photon energy in colloidal clay systems. Appl. Clay Sci..

[7] Ishida Y., Kulasekharan R., Shimada T., Ramamurthy V., Takagi S. (2014). Supramolecular-Surface Photochemistry: Supramolecular Assembly Organized on a Clay Surface Facilitates Energy Transfer between an Encapsulated Donor and a Free Acceptor. J. Phys. Chem. C.

[8] Kloprogge J.T., Hartman H. (2022). Clays and the Origin of Life: The Experiments. Life.

[9] Sato H., Takimoto K., Kato M., Nagaoka S., Tamura K., Yamagishi A. (2020). Real-Time Monitoring of Low Pressure Oxygen Molecules over Wide Temperature Range: Feasibility of Ultrathin Hybrid Films of Iridium(III) Complexes and Clay Nanosheets. Bull. Chem. Soc. Jpn..

[10] Okada T., Watanabe Y., Ogawa M. (2005). Photoregulation of the intercalation behavior of phenol for azobenzene–clay intercalation compounds. J. Mater. Chem..

[11] Ramamurthy V., Sivaguru J. (2016). Supramolecular Photochemistry as a Potential Synthetic Tool: Photocycloaddition. Chem. Rev..

[12] Liu Robert S.H., Hammond G.S. (2005). Reflection on Medium Effects on Photochemical Reactivity. Acc. Chem. Res..

[13] Li S., Mu B., Wang X., Wang A. (2021). Recent researches on natural pigments stabilized by clay minerals: A review. Dye. Pigment..

[14] Kohno Y., Kinoshita K., Ikoma S., Yoda K., Shibata M., Matsushima R., Tomita Y., Maeda Y., Kobayashi K. (2009). Stabilization of natural anthocyanin by intercalation into montmorillonite. Appl. Clay Sci..

[15] Kohno Y., Totsuka K., Ikoma S., Yoda K., Shibata M., Matsushima R., Tomita Y., Maeda Y., Kobayashi K. (2009). Photostability enhancement of anionic natural dye by intercalation into hydrotalcite. J. Colloid Interface Sci..

[16] Teepakakorn A.P., Bureekaew S., Ogawa M. (2018). Adsorption-Induced Dye Stability of Cationic Dyes on Clay Nanosheets. Langmuir.

[17] Ishii A., Itoh T., Kageyama H., Mizoguchi T., Kodera Y., Matsushima A., Torii K., Inada Y. (1995). Photostabilization of Chlorophyll a Adsorbed onto Smectite. Dye. Pigment..

[18] Saito T., Fukui K., Kodera Y., Matsushima A., Nishimura H., Inada Y. (2005). Photostability of biliverdin bound to smectite, clay mineral. Dye. Pigment..

[19] Cheng M., Song W., Ma W., Chen C., Zhao J., Lin J., Zhu H. (2008). Catalytic activity of iron species in layered clays for photodegradation of organic dyes under visible irradiation. Appl. Catal. B Environ..

[20] Feng J., Hu X., Yue P., Zhu H., Lu G. (2003). Degradation of Azo-dye Orange II by a Photoassisted Fenton Reaction Using a Novel Composite of Iron Oxide and Silicate Nanoparticles as a Catalyst. Ind. Eng. Chem. Res..

[21] Cheng M., Ma W., Chen C., Yao J., Zhao J. (2006). Photocatalytic degradation of organic pollutants catalyzed by layered iron(II) bipyridine complex–clay hybrid under visible irradiation. Appl. Catal. B Environ..

[22] Tani S., Yamaki H., Sumiyoshi A., Suzuki Y., Hasegawa S., Yamazaki S., Kawamata J. (2009). Enhanced Photodegradation of Organic Dyes Adsorbed on a Clay. J. Nanosci. Nanotechnol..

[23] Ishida Y., Masui D., Shimada T., Tachibana H., Inoue H., Takagi S. (2012). The Mechanism of the Porphyrin Spectral Shift on Inorganic Nanosheets: The Molecular Flattening Induced by the Strong Host–Guest Interaction due to the “Size-Matching Rule”. J. Phys. Chem. C.

[24] Tsukamoto T., Shimada T., Takagi S. (2013). Unique Photochemical Properties of p-Substituted Cationic Triphenylbenzene Derivatives on a Clay Layer Surface. J. Phys. Chem. C.

[25] Ishida Y., Shimada T., Takagi S. (2014). “Surface-Fixation Induced Emission” of Porphyrazine Dye by a Complexation with Inorganic Nanosheets. J. Phys. Chem. C.

[26] Tsukamoto T., Shimada T., Takagi S. (2015). Structure resembling effect of clay surface on photochemical properties of meso-phenyl or pyridyl-substituted monocationic antimony(V) porphyrin derivatives. RSC Adv..

[27] Umemoto T., Ohtani Y., Tsukamoto T., Shimada T., Takagi S. (2014). Pinning effect for photoisomerization of a dicationic azobenzene derivative by anionic sites of the clay surface. Chem. Commun..

[28] Arakawa K., Shimada T., Ishida T., Takagi S. (2021). “In-water” Dehydration Reaction of an Aromatic Diol on an Inorganic Surface. Langmuir.

[29] Takagi S., Shimada T., Ishida Y., Fujimura T., Masui D., Tachibana H., Eguchi M., Inoue H. (2013). Size-Matching Effect on Inorganic Nanosheets: Control of Distance, Alignment, and Orientation of Molecular Adsorption as a Bottom-Up Methodology for Nanomaterials. Langmuir.

[30] Takagi S., Shimada T., Eguchi M., Yui T., Yoshida H., Tryk D.A., Inoue H. (2002). High-Density Adsorption of Cationic Porphyrins on Clay Layer Surfaces without Aggregation: The Size-Matching Effect. Langmuir.

[31] Tsukamoto T., Shimada T., Shiragami T., Takagi S. (2015). Photochemical Chlorination and Oxygenation Reaction of Cyclohexene Sensitized by Ga(III) Porphyrin–Clay Minerals System with High Durability and Usability. Bull. Chem. Soc. Jpn..

[32] Golec B., Buczyńska J., Nawara K., Gorski A., Waluk J. (2023). Photodegradation of free base and zinc porphyrins in the presence and absence of oxygen. Photochem. Photobiol. Sci..

[33] Nakayama A., Mizuno J., Ohtani Y., Shimada T., Takagi S. (2018). Elucidation of the Adsorption Distribution of Cationic Porphyrin on the Inorganic Surface by Energy Transfer as a Molecular Ruler. J. Phys. Chem. C.

[34] Ishida Y., Shimada T., Tachibana H., Inoue H., Takagi S. (2012). Regulation of the Collisional Self-Quenching of Fluorescence in Clay/Porphyrin Complex by Strong Host–Guest Interaction. J. Phys. Chem. A.

[35] Silva A.M.S., Neves M.G.P.M.S., Martins R.R.L., Cavaleiro J.A.S., Boschi T., Tagliatesta P. (1998). Photo-oxygenation of meso-tetraphenylporphyrin derivatives: The influence of the substitution pattern and characterization of the reaction products. J. Porphyr. Phthalocyanines.

[36] Ramamurthy V., Mondal B. (2015). Supramolecular photochemistry concepts highlighted with select examples. J. Photochem. Photobiol. C Photochem. Rev..

