# Supplementary material for: Effects of Clay Nanosheets on the Photostability of Cationic Porphyrin
Source: Molecules. 2024 Aug 7;29(16):3738. doi: 10.3390/molecules29163738 (PMC11357654; doi:10.3390/molecules29163738)
Supplement: Supplementary file 1 [file molecules-29-03738-s001.zip › molecules-3108021-supplementary.pdf]

Supporting information

# Effects of Clay Nanosheets on the Photostability of Cationic Porphyrin

**Yoshinori Tahara [1], Yugo Hirade [2], Kyosuke Arakawa [3], Tetsuya Shimada [1], Tamao Ishida [1,4], Hiroshi Tachibana [1] and Shinsuke Takagi [1,4,*]**

[1] Department of Applied Chemistry, Faculty of Urban Environmental Sciences, Tokyo Metropolitan University, Hachiohji 192-0397, Tokyo, Japan
[2] Advanced Collaborative Research Organization for Smart Society (ACROSS), Waseda University, Shinjuku-ku 169-8555, Tokyo, Japan
[3] Department of Applied and Pure Chemistry, Faculty of Science and Technology, Tokyo University of Science, Noda-City 278-8510, Chiba, Japan
[4] Research Center for Hydrogen Energy-Based Society (ReHES), Tokyo Metropolitan University, Hachiohji 192-0397, Tokyo, Japan

* Correspondence: takagi-shinsuke@tmu.ac.jp

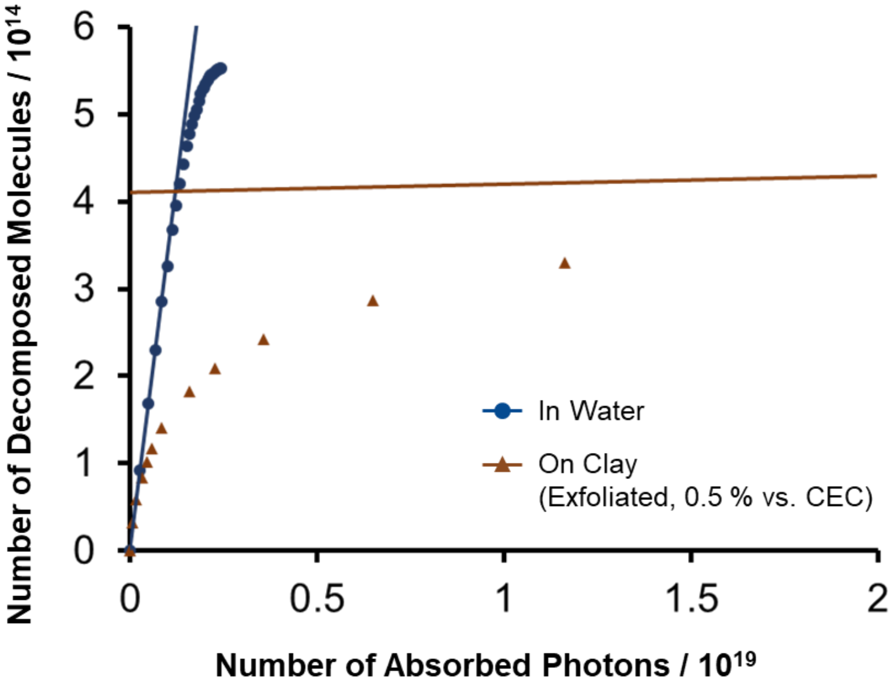


**Figure S1.** Photodecomposition behavior of $ZnTMAP^{4+}$ in water and in exfoliated clay dispersion.
